# Elapsed time between ICU request and actual admission of patients with SIRS/sepsis leads to an increase in mortality or length of stay in this unit?

**DOI:** 10.1186/cc12956

**Published:** 2013-11-05

**Authors:** Edmilson Bastos de Moura, Fábio Ferreira Amorim, Adriell Ramalho Santana, Jaqueline Lima de Souza, Felipe Bozi Soares, Bárbara Magalhães Menezes, Mariana Pinheiro Barbosa de Araújo, Fernanda Vilas Bôas Araújo, Louise Cristhine de Carvalho Santos, Pedro Henrique Gomes Rocha, Guilherme Menezes de Andrade Filho, Thiago Alves Silva, Pedro Nery Ferreira Júnior, Alethea Patrícia Pontes Amorim, José Aires de Araújo Neto, Marcelo de Oliveira Maia

**Affiliations:** 1 Hospital Santa Luzia, Brasília, Brazil; 2 Escola Superior de Ciências da Saúde, Brasília, Brazil; 3 Liga Acadêmica de Medicina Intensiva de Brasília, Brazil

## Background

Measures to ensure an appropriate early treatment for critically ill patients result in significant decreases in mortality [1,2]. This study aims to evaluate the impact of the elapsed time between ICU request and actual admission of patients with SIRS/sepsis on ICU mortality and length of stay.

## Materials and methods

Retrospective cohort study conducted in the ICU of Hospital Santa Luzia, Brasilia, DF, during 3 months. Patients being consecutively admitted to the ICU with diagnostic of SIRS/sepsis were divided into two groups: those with elapsed time between ICU request and admission less than 6 hours (short waiting period group (SWP)) or over 6 hours (long waiting period group (LWP)).

## Results

A total of 70 patients were enrolled (46% of admissions), 14 patients with SIRS, 27 with sepsis, 13 with severe sepsis and 17 with septic shock. For the entire cohort, the mean age was 61 ± 22 years, APACHE II was 12 ± 7.7, ICU length of stay was 15 ± 22.8 days, and 39 were male (54,9%). Thirty-five patients belonged to the LWP (50%). LWP patients had higher mortality (50% vs. 19.6%, *P *= 0.04), and longer ICU length of stay (13.6 ± 18.5 vs. 23.5 ± 40.7 days, *P *= 0.04). Relative risk for death in the LWP was 2.83 (95% CI: 1.28 to 6.28).

## Conclusions

The elapsed time between ICU request and actual admission of patients with SIRS/sepsis over 6 hours resulted in increased ICU mortality and ICU length of stay for this group of patients.
